# NICE Cancer Guidance: a description of the Institute's Clinical Guideline Programme

**DOI:** 10.1038/sj.bjc.6601078

**Published:** 2003-08-15

**Authors:** P Littlejohns, M Eccles, G Leng

**Affiliations:** 1 National Institute for Clinical Excellence, 11 Strand, London WC2N 5HR, UK

**Keywords:** healthcare system, oncology care, clinical practice guidelines

The National Institute for Clinical Excellence (NICE) was established to improve the quality of health care in England and Wales through the provision of guidance on the clinical and cost-effectiveness of health interventions.

The treatment of cancer is one of the government's priority areas and a range of guidance products have been developed by the Institute to support implementation of national cancer initiatives. Out of a total of 56, 15 technology appraisals issued in the first 3 years of the Institute's existence are related to the general area of oncology, with another four in preparation. Two service guidance documents on breast and urological cancer have been issued, with six in progress. Clinical guidelines on lung cancer and familial breast cancer have been commissioned and the Department of Health's referral guidance for patients with suspected cancer is being updated.

This paper describes how the Institute develops the clinical guidelines component of its guidance portfolio.

## THE ROLE OF THE INSTITUTE

NICE was established as a special Health Authority in April 1999. It has four main aims:

(I) to speed up the uptake by the National Health Service (NHS) of interventions that are both clinically and cost effective;
(II) to encourage more equitable access to healthcare (i.e. ‘reduce the postcode lottery of care’);
(III) to provide better and more rational use of available resources by focusing the provision of health care on the most cost-effective interventions; and
(IV) to encourage the creation of new and innovative technologies.

The Institute achieves these aims by providing guidance to the NHS on the effectiveness and cost-effectiveness of clinical interventions. This task is achieved by appraising new and existing technologies and developing disease-specific clinical guidelines. In addition, the Institute has responsibility for the four national confidential enquiries and more recently for assessing the safety and efficacy of new interventional procedures (previously the responsibility of SERNIP–Safety and Efficacy Register of New Interventional Procedures) and advising on the safety of borderline substances. The previous work of the Cancer Clinical Outcomes Group on service guidance to support the implementation of the Calman Hine report (see separate editorial) and the cancer referral guidance are also now part of the NICE portfolio of guidance (Table 1 shows the Institute's products in the field of cancer care).

In its first 3 years, the Institute's main output has been in the technology appraisals programme and this has created considerable interest and some controversy, particularly in the area of oncology. The explicit approach that the Institute has adopted in reaching its decisions has highlighted difficult ethical issues that need to be addressed in seeking to balance the desires of individuals with population health requirements. Details of how the Institute is responding to these challenges is documented elsewhere (Littlejohns *et al*, 2003).

## QUALITY ASSURANCE

Politicians have given strong messages about the need to improve quality within the NHS and have developed a ‘quality improvement’ model with education and support being key driving forces. The various institutions involved in this developing quality initiative, NICE, the Modernisation Agency (Cancer Services Collaborative), the Commission for Health Improvement, the National Clinical Governance Support Team, and more recently the National Patient Safety Agency, have indicated that their role is to support the NHS in addressing the daily challenges of delivering high-quality health care. This is particularly important when expectations and demand frequently exceed what is feasible within current service configurations.

The Institute's contribution was first outlined in the white papers, entitled ‘The new NHS: modern and dependable’ and ‘A first class service: quality in the new NHS’ (Department of Health, 1997,^1998^). Standards for service configuration are established at a national level through the creation of national service frameworks (in the context of cancer services, the national plan) and clinical standards are established through the guidance issued by NICE.

In this context, the Institute considers that a key role is to provide guidance on controversial health issues where lack of clarity has resulted in regional variation in the care provided by the NHS. The guidance produced is expected to be incorporated into local clinical governance mechanisms (including cancer networks) via the use of local guidelines and protocols.

## THE CLINICAL GUIDELINE PROGRAMME

NICE produces clinical guidelines (defined as systematically developed statements to assist practitioner and patient decisions about appropriate health care for specific clinical circumstances that cover all aspects of patient care from prevention and self-care through primary and secondary care to more specialised services (Field and Lohr, 1990).

In the past, while there have been many professional groups and organisations producing guidelines, these were of variable quality and none addressed cost-effectiveness (Grilli *et al*, 2000). A guideline development programme has been designed that is evidence based, consultative, transparent and inclusive, and adheres to internationally recognised standards of practice (The AGREE Collaboration, 2003). Details of the process are described in a series of manuals available at the following Internet address: http://www.nice.org.uk/cat.asp?c=22334. The principles underlying the Institute's clinical guidelines are given in Table 2.

The main role of the Institute is to commission, coordinate and quality assure the process. To achieve this, it relies on its commissioning managers and the Guidelines Advisory Committee (to be reconfigured as guideline review panels from 1st April 2003). The members provide external validation for the guidelines by overseeing the development process, advising on the commissioning of work and monitoring the quality of the guidelines. These members have a range of backgrounds and knowledge, including guideline expertise, clinical experience, patient/carer involvement and health economics.

## THE NATIONAL COLLABORATING CENTRES AND THE SUPPORT UNITS

Six professionally led National Collaborating Centres (NCCs) have been established. A seventh collaborating centre specialising in cancer guidance has recently been commissioned. Each centre is a multidisciplinary collaboration of professionals, user representatives and technical experts.

They are expected to have access to a range of skills and abilities, either via an academic unit or by employing appropriate staff. These include clinical networks to support the proposed range of activities, expertise in engaging with patients and patient groups and methodological skills for guideline development. In addition to the NCCs, the Institute has established two support units to undertake guideline methodological research and assist patients to participate actively in the guideline development process.

## GUIDELINE TOPICS

The Department of Health for England and the Welsh Assembly Government select topics for the Institute's work programme. Topics are selected on the basis of whether a guideline would:

- have significant health benefits (e.g. reduce illness, disability or premature death);
- have a significant impact on NHS resources;
- complement one or more of the Institute's technology appraisals;
- link with key Government priorities for health; and
- help to resolve unacceptable variation in practice across England and Wales.

## GUIDELINE DEVELOPMENT PROCESSES

Each clinical guideline is developed in a process comprising seven phases: initiation, scoping, elaboration of a work plan, development, validation, publication and dissemination, review and update.

### Initiation

The Department of Health and the Welsh Assembly Government formally refer the guidelines work programme to the Institute. Once referred, notification of the programme is given through a press release and via the Institute's web site. A topic is allocated to a National Collaborating Centre, which begins the scoping exercise for the guidelines producing a provisional timetable for the work programme, drawing up timelines and identifying potential stakeholders for each guideline. A provisional timetable and details of the work programme are posted on the Institute's web site.

For the purposes of the Institute's clinical guideline development, stakeholders are:

- the national patient and carer organisations that represent people whose care is described in the guideline;
- the national organisations that represent the healthcare professionals who are directly providing the services described in the guideline;
- the companies or manufacturers of the medicines or devices used in the clinical area covered by the guideline and whose interests are may be significantly affected by the guideline;
- a number of primary care organisations and acute trusts–these organisations are invited to act as NHS stakeholders for a particular guideline; and
- the Department of Health and Welsh Assembly Government.

### Scoping

The scoping document is then drawn up. It describes the aspects of care that the guideline will cover, the background epidemiology, the population, the healthcare setting, and inclusions and exclusions of interventions and treatments. This goes through a period of external consultation following which the final scope is posted on the Institute's web site.

During the scoping phase, there is a meeting with all stakeholder organisations. In addition, the Patient Involvement Unit arranges a meeting for patient/carer stakeholder organisations to describe the opportunities for them to contribute to guideline development (including opportunities to nominate patient/carer representatives to sit on the Guideline Development Group) and to discuss the scope of the guideline and what this might mean to patients and carers.

### Elaboration of a work plan

Following agreement of the scope with the Collaborating Centre, the Institute works with the NCC to prepare a detailed work plan. This will follow a standard template and includes information on costs, timelines, methodology and the proposed membership of the Guideline development Group.

### Development

The Collaborating Centre is responsible for the development of the guideline through the establishment of the Guideline Development Group, with membership capable of considering and interpreting the evidence presented to it and formulating recommendations on appropriate clinical practice.

The membership of the Group should reflect the range of clinical disciplines involved in providing care and should include patient/carer representatives. The facilitator/chairman is selected by the NCC, in agreement with the Institute.

The key stages in the development of the Institute's guidelines are described in detail in Chapter 3 of the Health Technology Assessment monograph ‘How to Develop Cost-conscious Guidelines’ (Eccles and Mason, 2001). Also, a technical manual building on the first 2 years' experiences of the NCCs is being compiled. Key stakeholders are also invited to list information for consideration by the National Collaborating Centre, and highlight appropriate data that will become available during the development phase.

The Institute's guidelines are expected to address cost effectiveness as well as clinical effectiveness. There are a number of possible ways in which economic methods can be used in the production of clinical guidelines, such as profiling, modelling, cost–impact assessment, and using literature reviews of economic analyses. The guideline development process incorporates a health economist whose prime role is to argue for full discussion and useful presentation of the value of treatment alternatives.

All commissioned guidelines are subject to a period of implementation planning with local health communities (geographical clusters of one or more primary- and secondary-care providers in England and Wales).

### Validation

Three versions of the guideline are produced based on the recommendations of the National Collaborating Centre (Table 3). These go through a two-stage consultation process, which offers an opportunity for wide consultation on the drafts. Within this process, stakeholders comment on the draft guideline and the developers respond to all received comments. Following this consultation, the redrafted guideline, and all the comments and responses are considered by a subgroup of the Guidelines Advisory Committee. In response to their advice the final draft of the full guideline, short-form guideline and patient version are prepared by the Collaborating Centre. The Chair of the sub-group signs off the final draft of the full guideline on behalf of the Guidelines Advisory Committee and submits it to the Institute. Following approval, the Institute signs off the final draft of the full guideline and returns the final draft to the Collaborating Centre for publication.

### Publication and dissemination

The short version guideline and the patient version are published by the Institute. Copies of the full version of the guideline are available from the Collaborating Centre.

### Review and update

When the Institute publishes the guideline, a date is given on which the guidance will be reviewed. The length of time between the issue of the guideline and the review date varies depending on the anticipated rate of change in the evidence for the guideline.

## CONCLUSIONS

This is an ambitious programme. The wide range of its portfolio makes the Institute's guidance programme among the largest in the world. Its sustainability will depend on liaising with many professional and patient organisations within the UK as well as initiating international collaborations. The Institute is looking forward to contributing to a European-wide approach to developing cancer guidelines (Philip *et al*, 2003). NICE'S responsibility for providing guidance on cost-effectiveness as well as clinical effectiveness will ensure that it will continue to be controversial and to challenge the *status quo*.

## References

[bib1] AGREE Collaboration (2003) Development and validation of an international appraisal instrument for assessing the quality of clinical practice guidelines: the AGREE project. Qual Saf Health Care, 12: 18–23 12571340 10.1136/qhc.12.1.18 PMC1743672

[bib2] Department of Health (1997) The New NHS: Modern and Dependable. HMSO: London

[bib3] Department of Health (1998) A First Class Service: Quality in the New NHS. HMSO: London

[bib4] Eccles M, Mason J (2001) How to develop cost-conscious guidelines. Health Technol Assess 5: 1–69 10.3310/hta5160 11427188

[bib5] Field MJ, Lohr KN (eds) (1990) Clinical Practice Guidelines: Directions for a New Programme. National Academy Press: Washington, DC 25144032

[bib6] Grilli R, Magrini N, Penna A, Mura G, Liberati A (2000) Practice guidelines developed by specialty societies. The need for a critical appraisal. Lancet 355: 103–106 10675167 10.1016/S0140-6736(99)02171-6

[bib7] Littlejohns P, Barnett D, Longson C (2003) The Cancer Technology Appraisal Programme of the National Institute for Clinical Excellence. Lancet Oncol 4: 242–250 12681268 10.1016/s1470-2045(03)01036-2

[bib8] Philip T, Fervers B, Haugh M, Browman G (2003) European co-operation for clinical practice guidelines in cancer. Br J Cancer 89(Suppl 1): S1–S3 10.1038/sj.bjc.6601076 PMC2753000 12915895

