# NICE Cancer Guidance: a description of the Institute's Clinical Guideline Programme

**DOI:** 10.1038/sj.bjc.6601408

**Published:** 2003-11-25

**Authors:** P Littlejohns, M Eccles, G Leng

## Abstract

**Correction to:** *British Journal of Cancer* 2003; **89** (S1), S9–S11 doi:10.1038/sj.bjc.6601078

Unfortunately, due to an error, the tables were omitted from the above article.

All the three tables (Table 1

**Table 1:** Range of NICE guidance affecting patients with cancer

*Technology appraisals*
Drugs (Paclitaxel, Docetaxel, Trastuzumab, Vinorelbine, Topetecan, Caelyx, Temozolamide, Irintecan, Oxaliplatin, Raltitexed, Gemcitabine, Vinorelbine, Fludarabine, Imatinib)
Liquid-based cytology
Laparoscopic surgery for colorectal cancer

*Clinical guidelines*
Lung cancer
Familial breast cancer

*Cancer service guidances*
Breast, urology, haematico-oncological, colorectal, head and neck, child and adolescent, brain and cns, skin, supportive and palliative care

*Cancer referral guidance*

*Confidential enquiries*
Review of cancer care in acute units

, 2

**Table 2:** Key principles underlying the Institute's clinical guidelines

NICE clinical guidelines should:
•aim to improve the quality of clinical care
•address the clinical effectiveness of treatments or management approaches (that is, how well treatments or management approaches work)
•address the cost effectiveness of treatments or management approaches
•be advisory
•be developed through a process that considers all those who might be affected by the guideline (usually including healthcare professionals, patients and their carers, service managers, the wider public, Government and the healthcare industries)
•be based on the best possible research evidence and professional consensus
•be developed using methods that command the respect of patients, the NHS and NHS stakeholders
•be developed with the involvement of patients and healthcare professionals
•set out the clinical care that might reasonably be expected throughout the NHS in England and Wales

and 3

**Table 3:** The three versions of the clinical guideline

*1. NICE GUIDELINE (SHORT VERSION)*
This uses a standard template, and contains:
•the Institute's guidance to the NHS
•implementation advice
•audit advice
•information on the expected impact on NHS resources
•a review date
•a clinical practice algorithm
•details of the Guideline Development Group members

*2. NATIONAL COLLABORATING CENTRE GUIDELINE (FULL VERSION)*
This includes:
•the recommendations
•the profile of treatments and interventions assessed by the Guideline Development Group
•a description of how the evidence was collected, reviewed and assessed
•a description of how the recommendations were formulated and graded
•full reference details of the literature in the evidence base
•details of the Guideline Development Group members
•names of the stakeholders who commented on the first draft of the guideline

*3. INFORMATION FOR THE PUBLIC (PATIENT VERSION)*
This version:
•interprets the recommendations made in the NICE Guideline for patients and carers
•aims to help patients to make informed choices about their care
•can be reproduced in patient/carer organisations’ own material, with the permission of the Institute

) are reprinted below:

The publisher would like to apologise for any inconvenience this error may have caused.

